# Extraskeletal Chondroma: A Rare Cause of Trigger Finger in Children

**DOI:** 10.1155/2020/8259089

**Published:** 2020-01-06

**Authors:** Giada Salvatori, Caterina Novella Abati, Camilla Bettuzzi, Anna Maria Buccoliero, Chiara Caporalini, Alessandro Zanardi, Manuele Lampasi

**Affiliations:** ^1^Department of Paediatric Orthopaedics and Traumatology, Anna Meyer Children's University Hospital, Florence, Italy; ^2^Division of Pathological Anatomy, Anna Meyer Children's University Hospital, Florence, Italy

## Abstract

**Introduction:**

Trigger finger is ten times less common than trigger thumb in infants and children and, unlike trigger thumb, may arise from a variety of underlying causes. To our knowledge, we describe the first case of pediatric trigger finger secondary to an extraskeletal chondroma.

**Case Presentation:**

We report the case of an 11-year-old girl presenting with a typical history of triggering of the fourth finger, in whom a nodule attached to the flexor digitorum superficialis was found; clinical, ultrasound, and operative findings are described. Histological analysis was diagnostic of extraskeletal chondroma, also known as chondroma of soft tissues.

**Conclusion:**

This is a very uncommon benign cartilaginous tumor, mostly reported in patients aged 30 to 60 years (just one pediatric extraskeletal chondroma of the hand has been described), and presentation with trigger finger has been reported just once, in a 76-year-old man. This condition should be considered in the differential diagnosis of pediatric trigger finger.

## 1. Introduction

Trigger finger is ten times less common than trigger thumb in infants and children [1].

Unlike trigger thumb, pediatric trigger finger may arise from a variety of underlying anatomic causes, including nodularity or thickening of the flexor digitorum superficialis (FDS) or flexor digitorum profundus (FDP) tendons, abnormal relationships between the FDS and FDP tendons, proximal FDS decussation, and constriction of the A1, A2, or A3 pulleys. More uncommon causes include intratendinous calcification, granulation tissue, cysts, and the association with mucopolysaccharide storage disorders (Hurler syndrome and Hunter syndrome) [1].

To our knowledge, trigger finger secondary to an extraskeletal chondroma (EC) has never been reported in children.

The aim of this work was to describe our experience of a trigger finger in an 11-year-old girl, which was found to be secondary to EC.

## 2. Case Report

An 11-year-old girl presented with a typical history of triggering of the fourth finger of her left hand which started six months before. The girl could move completely her finger, but in the position of flexion, locking of the digit occurred and passive unlocking caused discomfort.

At physical examination, a nodule of about 5 mm was palpable at the site of triggering, just distally to the distal palmar crease in line with the fourth finger.

**Figure 1:**
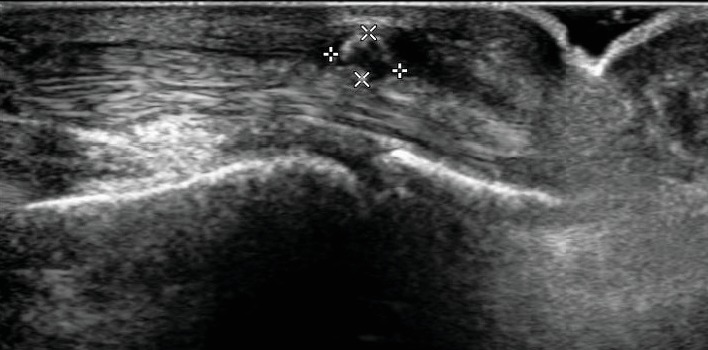
Ultrasound examination of the fourth finger of her left hand shows an avascular mass of about 6 × 2 mm, attached to the flexor tendons, anechogenic with an echogenic core.

Ultrasound examination (Figure 1) showed an avascular mass of about 6 × 2 mm, attached to the flexor tendons, anechogenic with an echogenic core.

**Figure 2:**
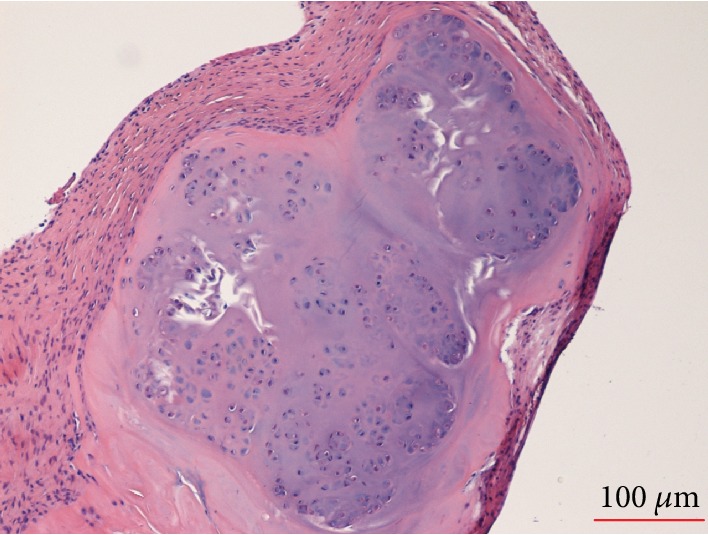
Histopathology. The resected specimen was an unorientable fragment of about 5 mm of diameter. It was routinely formalin fixed and paraffin embedded for the histological study. Microscopic examination revealed that the lesion consisted of three components: fibroblastic spindle cells, cartilage without significant cellular atypia, and small marginal irregular bony trabeculae. These features were diagnostic of chondroma.

**Figure 3:**
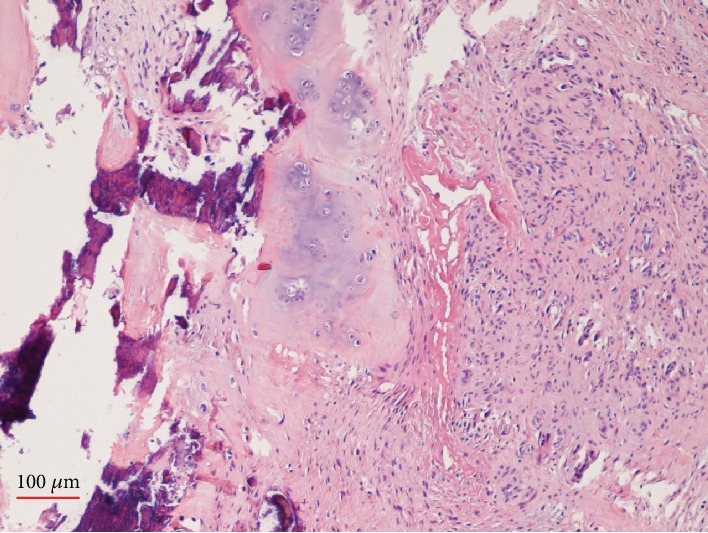
Histopathology.

Surgery for excisional biopsy and release of the triggering was performed under general anesthesia. A 1.5 cm transverse skin incision was performed at the distal palmar crease, just proximally to the A1 pulley area. Longitudinal incision of the A1 pulley revealed the nodule with a diameter of about 6 mm, attached to the tendon of the FDS. The nodule moved out of the A1 pulley as the digit was flexed and entered the pulley with difficulty as the digit was extended, causing catching and sudden release of the tendon. No relationship with the FDP or with the underlying metacarpal bone was found. The mass was excised from the tendon using a scalpel and referred for histopathological examination. The histological analysis (Figures 2 and 3) was diagnostic of chondroma.

The postoperative course was uneventful with rapid recovery of the complete range of motion. At the last follow-up, 27 months after surgery, magnetic resonance did not show recurrence and the patient did not report any episode of triggering after the operation.

## 3. Discussion

EC (or soft tissue chondroma) is a rare benign (1.5% of all benign soft tissue tumors) cartilaginous tumor with uncertain aetiology [2] arising from soft tissues such as tendons, tendon sheath synovia, and joint capsules with no continuity to the bone or periosteum and with tendency to occur in the hands and feet. It affects both sexes equally and mainly occurs in patients aged 30 to 60 years [3].

First described by Baumuller in 1883 [2], EC is a distinct histopathological diagnosis in contrast to other cartilage-containing lesions and characterized by cellular atypism. Areas of ossification and calcification may be found within the hyaline cartilage that composes most of the lesion [2].

Our literature analysis of EC affecting the hand and upper extremity (Table 1) revealed 25 cases reported [2, 4–16], located in digits (eight cases), within the carpal tunnel (three), or in other sites in the hand (ten); in four cases, information about the exact location was missing. The average size ranged from 0.5 to 12 cm in an estimated diameter. There was clear male predominance. The average patient age was 46 years, but age was not reported in all instances. Just one pediatric case was reported, a 12-year-old male patient presenting with a history of enlarging mass over the hypothenar eminence of the hand [5].

Clinical appearance was typically characterized by swelling due to a palpable subcutaneous mass; the three cases within the carpal tunnel were associated with symptoms of carpal tunnel syndrome. Trigger finger secondary to EC, similar to what we have reported in an 11-year-old girl, was described only in a 76-year-old man [2]. All cases underwent surgical excision with a recurrence rate reported to be 15% to 25% [2].

## 4. Conclusion

Extraskeletal chondroma is a rare benign tumor in the hand. Depending on where the EC is located, it can cause symptoms and mimic other conditions as described in our case [2]. This condition should be considered in the differential diagnosis of pediatric trigger finger.

## Figures and Tables

**Table 1:** Review of the published cases of EC affecting the hand and upper extremity.

Author	Year of publication	Number of cases	Exact site of origin	Therapy	Patient age (y)	Gender	Size of chondroma (cm)
Wenny et al. [16]	2018	1	Left hand	Surgical excision	54	M	4 × 7
Saito et al. [4]	2017	1	Index finger	Surgical excision	63	M	4 × 3.8
Schwaiger et al. [2]	2017	1	Ring finger	Surgical excision	76	M	3 × 1 × 1
Khandeparkar et al. [6]	2014	1	Radial aspect of the wrist	Surgical excision	52	M	5 × 4 × 3
Ikeda and Osamura [7]	2013	1	Carpal tunnel	Surgical excision	82	F	12 × 4.5 × 4.2
Suganuma et al. [3]	2011	1	Index finger	Surgical excision	62	M	5 × 4.5 × 5
Ishii et al. [8]	2010	1	Index finger, subungual	Surgical excision	39	M	—
Le Corroller et al. [9]	2008	1	Palmar side of the distal forearm	Surgical excision	40	M	Diameter 4.5
Singh et al. [5]	2005	1	Hypothenar eminence	Surgical excision	12	M	4 × 4
Cumming et al. [10]	2005	1	Carpal tunnel	Surgical excision	47	M	—
De Smet 11	2005	1	Elbow/proximal forearm	Surgical excision	50	M	Diameter 3-4
Cho and Kim 12	2003	1	Ring finger, subungual	Surgical excision	21	M	Diameter 0.5
Boudart et al. [13]	2003	1	Carpal tunnel	Surgical excision	18	F	—
Thool et al. [14]	2001	1	Forearm	Surgical excision	54	M	6 × 5
De Poulpiquet et al. [15]	1999	1	Little finger	Surgical excision	26	M	—
Nakamura et al. [2]	1997	—	—	—	—	—	—
Yamada et al. [2]	1995	1	Ring finger	Surgical excision	51	M	4 × 2.2 × 1.5
Isayama et al. [2]	1991	1	Thumb	Surgical excision	44	M	—
DelSignore et al. [2]	1990	1	Palmar side of the hand	Surgical excision	—	—	—
Van Demark et al. [2]	1990	—	—	—	—	—	—
Marcial-Seoane et al. [2]	1990	1	Upper arm	Surgical excision	—	—	—
Catalano et al. [2]	1988	—	—	—	—	—	—
Sowa et al. [2]	1987	2	Palmar and dorsal sides of the hand	Surgical excision	—	—	—
Perri and Tripi [2]	1986	1	Wrist	Surgical excision	—	—	—

## Additional Points

This study was conducted at the Anna Meyer Children's University Hospital, Florence, Italy.
